# A novel curcumin analog sAc15 alleviates LPS-induced acute lung injury by activating the PI3K/AKT/Nrf2 pathway in pulmonary epithelial cells

**DOI:** 10.1080/13510002.2026.2721883

**Published:** 2026-08-22

**Authors:** Liqin Zhou, Zhiyi Tang, Yuting Lin, Tengfei Zhou, Mengya Shen, Yuxi Lin, Chengshui Chen, Chaolei Chen

**Affiliations:** a Department of Pulmonary and Critical Care Medicine, Zhejiang Provincial Key Laboratory of Interventional Pulmonology, The First Affiliated Hospital of Wenzhou Medical University, Wenzhou, China; b Department of Physiology, Zhejiang University School of Medicine, Hangzhou, China; c Department of Pharmacy, Jiaxing Maternity and Child Health Care Hospital, Affiliated Women and Children's Hospital of Jiaxing University, Jiaxing, China; d Department of Pulmonary and Critical Care Medicine, The Quzhou Affiliated Hospital of Wenzhou Medical University, Quzhou People's Hospital, Quzhou, China

**Keywords:** Acute lung injury, curcumin analog, oxidative stress, epithelial cells, mitochondrial dysfunction, apoptosis, Nuclear factor erythroid 2-related factor 2 (Nrf2), PI3K/AKT pathway

## Abstract

**Objectives:**

Acute lung injury (ALI) is driven by oxidative stress and disrupted redox homeostasis. While curcumin is a potent natural antioxidant, its clinical application is limited. We evaluated the protective capacity and underlying mechanisms of sAc15, a stable curcumin analog, in ALI models.

**Methods:**

*In vivo*, mice were intratracheally instilled with lipopolysaccharide (LPS) to induce ALI. *In vitro*, Beas-2b cells were exposed to *t*-BHP for oxidative damage. Target engagement was evaluated via surface Plasmon resonance (SPR). Protective effects and mechanisms were evaluated via histopathology, ROS accumulation, apoptosis assays, network pharmacology, Western blotting, LY294002 and Nrf2-specific siRNA.

**Results:**

sAc15 exhibited broader safety margins and superior ROS suppression compared to natural curcumin. SPR established direct binding between sAc15 and recombinant Nrf2 (KD = 2.68 μM). *In vivo and in vitro*, sAc15 mitigated pulmonary inflammation, oxidative stress, mitochondrial dysfunction and apoptosis. Mechanistically, sAc15 promoting PI3K/AKT phosphorylation and upregulating the expression of Nrf2, HO-1 and NQO-1. Crucially, Nrf2 silencing or PI3K inhibition markedly attenuated sAc15-mediated cytoprotective, anti-apoptotic, and antioxidant benefits.

**Conclusion:**

sAc15 ameliorates ALI by activating the PI3K/AKT/Nrf2 axis to restore oxidative equilibrium, hightlighhting its promise as a therapeutic candidate for ALI/ARDS.

## 1. Introduction

Acute lung injury (ALI), first defined in 1967, is currently classified within the spectrum of mild to moderate acute respiratory distress syndrome (ARDS) [1,2]. Clinically, ALI predominantly manifests as acute hypoxemic respiratory failure resulting from diffuse pulmonary inflammation and edema [3]. Although the concept of ALI has been recognized for over five decades, therapeutic strategies have been extensively investigated only in the past two decades [2]. Among these, lung-protective ventilation strategies represent a significant advancement in ARDS management, and newer approaches such as airway pressure-release ventilation have demonstrated mortality benefits, albeit with considerable heterogeneity in treatment outcomes [4]. While corticosteroids and neuromuscular blockade are recommended in the American Thoracic Society clinical practice guidelines, optimal treatment protocols remain unclear [5]. Moreover, several novel therapies, including inhaled nitric oxide, surfactants, and mesenchymal stromal cell therapy, have yet to be established as standard clinical treatments [6–8]. Consequently, identifying effective therapeutic interventions for ALI remains an urgent clinical need.

The pathogenesis of ALI involves complex and overlapping activation and dysregulation of injury response pathways [9]. A hallmark feature is the vicious cycle of excessive inflammation and oxidative stress, leading to diffuse alveolar damage. In the early phase of injury, neutrophil migration across the epithelium results in the release of large amounts of reactive oxidants, directly causing oxidative epithelial damage [10]. The integrity of the lung epithelium, which functions as a critical barrier against pathogens, is a key determinant of survival in ARDS patients [11]. Damage to the epithelium leads to leakage of cellular contents, shedding of the pulmonary epithelial glycocalyx, and release of tissue factor, all of which further amplify inflammatory responses [12–14]. Therefore, therapeutic strategies aimed at mitigating oxidative damage-induced lung epithelial cell death are highly desirable.

Curcumin, a natural biphenolic compound extracted from turmeric rhizomes, exhibits multiple biological activities including antioxidant, anti-inflammatory, and antitumor effects [15]. However, the unstable *β*-diketone moiety in its structure results in poor stability and low bioavailability, hindering significant progress in clinical applications [16,17]. In our previous study, we synthesized a series of monoenone-monocarbonyl curcumin analogs (sAc) by structurally modifying the unstable *β*-diketone fraction through removal of the alkenyl double bond, a benzene ring, and a carbonyl group. These modifications improved compound stability and reduced toxicity, while preserving antioxidant properties. Notably, the analog sAc15 demonstrated superior activity and conferred protection against cerebral ischemia-reperfusion injury in murine models [18]. However, whether sAc15 exerts similar protective effects in ALI and the underlying molecular mechanisms remain unexplored.

In the present study, we employed lipopolysaccharide (LPS) to establish a murine model of ALI and used tert-butyl hydroperoxide (*t*-BHP) to induce oxidative injury in lung epithelial cells in vitro. We aimed to investigate the protective effects of sAc15 against acute lung injury and elucidate its potential mechanisms of action.

## 2. Materials and methods

### 2.1. Formulation of sAc15 and curcumin

**Figure 1.**
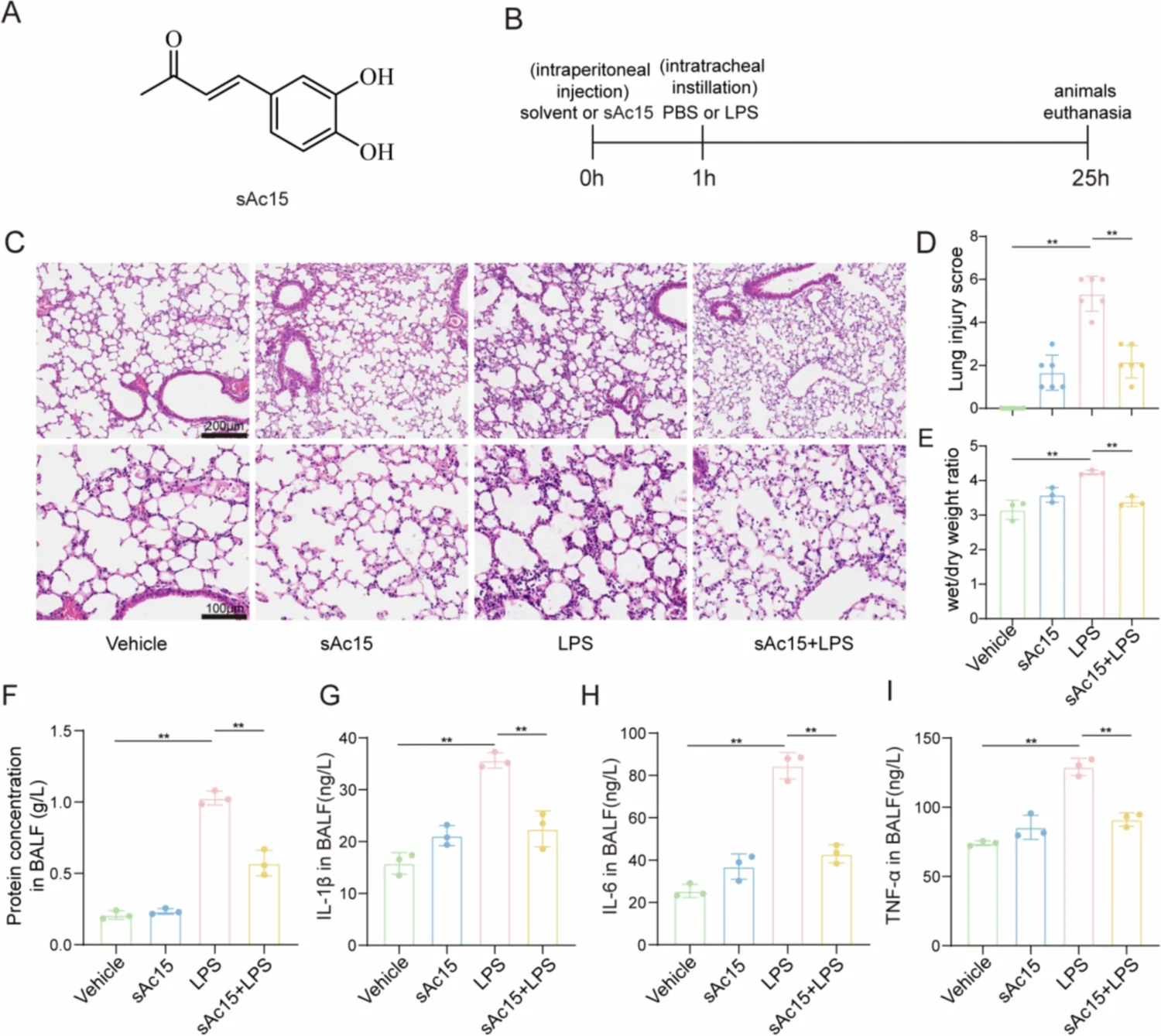
sAc15 attenuates LPS-induced ALI in mice. (A) Chemical structure of sAc15. (B) Experimental design. Wild-type C57BL/6 mice were split in four groups: Vehicle, sAc15 (20 mg/kg, 1 hour prior to LPS), LPS (10 mg/kg, intratracheal instillation), and sAc15+LPS. (C) H&E staining was used to assess the pathological changes in mice lung. Scale: 200 μm and 100 μm. (D) Lung injury score. (E) Lung wet/dry weight ratio were used to detect the degree of tissue edema. (F) Total protein concentration in BALF was determined using a BCA protein assay kit. (G–I) Levels of proinflammatory cytokines in BALF were determined by ELISA assay. Quantitative data were shown as means ± SEM (*n* = 3–6). *P < 0.05, **P < 0.01.

The curcumin analog sAc15 [(E)-4-(3,4-dihydroxyphenyl)but-3-en-2-one] was synthesized in the School of Pharmacy, Wenzhou Medical University, as previously described by Wenfei He et al. (Figure 1A) [18]. In brief, sAc-type monocarbonyl curcumin analogs were obtained through a proline-catalyzed condensation of acetone with the corresponding aromatic aldehydes at room temperature, followed by purification using column chromatography, yielding products with typical isolated yields of 40%–71%. The structure of sAc15 was confirmed by NMR and MS, and its purity (>98%) was verified by HPLC prior to use in the present study.

To prepare a 20 mM stock solution for cell experiments, 4.64 mg of sAc15 powder, which has a molecular weight of 146 g/mol, was diluted in 1.62 ml of DMSO. For administration to mice, a 0.002 mg/ml composition was prepared by dissolving 30 mg of sAc15 powder in a solvent ratio of DMSO: Cremophor: normal saline = 1: 19: 80.

The curcumin powder was purchased from MCE Company (HY-N0005, U.S.A.) and dissolved in DMSO to prepare a 20 mM working solution.

### 2.2. Animal experimental design

Male C57BL/6 mice (*n* = 48, 20–30 g) were fed at the Laboratory Animal Center of Wenzhou Medical University by Beijing Viton Lever Laboratory Animal Technology Company (Beijing, China). After 7 days of acclimatization, ALI was induced. All animal experimental procedures were reviewed and approved by the Institutional Animal Care and Use Committee of the First Affiliated Hospital of Wenzhou Medical University, Wenzhou, China (approval No. WYYY-IACUC-AEC-2024-049). No formal a priori sample size calculation was performed. Group sizes were based on our previous studies using similar ALI models and on practical considerations. The mice were randomly separated into four groups: Vehicle, sAc15, LPS and sAc15 + LPS (*n* = 12). One hour before intratracheal instillation, each group of mice received an intraperitoneal injection of an equal volume of solvent and sAc15 (20 mg/kg). The mice were then administered tracheal instillation of LPS (10 mg/kg, HY-D1056, MCE Company) under 0.5% pentobarbital sodium anesthesia (50 mg/kg) to induce ALI, while the vehicle and sAc15 groups received an equivalent amount of PBS as a negative control. The dose of sAc15 (20  mg/kg in the in vivo mouse experiments and 10 μM in the cell experiments) was determined based on our previous studies. Preliminary trial experiments confirmed that 20 mg/kg of sAc15 could exert anti-inflammatory and antioxidant effects in mouse cerebral ischemia-reperfusion injury without causing significant systemic toxicity [18,19]. After 24 hours, mice were euthanized by an intraperitoneal overdose of 0.5% sodium pentobarbital, after which bronchoalveolar lavage fluid (BALF) and lung tissues were harvested (Figure 1B). Statement that no predefined inclusion or exclusion criteria were used and that no animals or data points were excluded.

### 2.3. Cell culture and treatment

The Beas-2b human bronchial epithelial cells (Cell Bank of the Chinese Academy of Science, Shanghai, China) were cultured at 37°C with 95% air and 5% CO2 in a medium consisting of DMEM/F12 (89%, Gibco, U.S.A.), FBS (10%, Gibco, U.S.A.), and penicillin/streptomycin (1%, Gibco, U.S.A.). For pretreatment experiments, cells were treated with sAc15 (10 μM) for 1 hour before exposure to 30 μM *t*-BHP (B802372, Macklin, China) for 24 hours. After then, cell viability, ROS levels, mitochondrial membrane potential (MMP), and apoptosis-related proteins were analyzed. For post-treatment experiments, sAc15 (10 μM) was added at 0, 1, 2, 3, or 4 h after the initiation of t-BHP exposure, and all groups were analyzed 24 hours after t-BHP administration. For pathway inhibition, cells were treated with LY294002 (5 μM, MCE, U.S.A.) for 1 hour before sAc15 treatment.

### 2.4. Lung injury scoring

Fresh mouse lung tissues were fixed in 4% paraformaldehyde, dehydrated, paraffin-embedded, sectioned at 5 μm, hematoxylin and eosin (H&E) stained, and photographed under a light microscope. Lung injury scores were referred to the American Thoracic Society Workshop Report [20,21].

### 2.5. Collection and detection of BALF

The BALF was obtained and detected following a standardized procedure. Initially, a midline neck incision was made in mice to expose and isolate the trachea. A small incision was made in the trachea to insert and secure a catheter. Subsequently, a syringe containing 1 ml of precooled PBS was used to fill the lungs, and the BALF was aspirated three times. The collected fluid underwent centrifugation at a temperature of 4°C and a rotational speed of 3000 rpm for a duration of 15 minutes, with the resulting supernatant utilized for further analysis. Protein concentrations in the BALF were quantified using a BCA assay kit (23227, Thermo Fisher Scientific, U.S.A.). The level of proinflammatory cytokines and glutathione (GSH) in BALF was assessed using a readily available enzyme immunoassay kit (Beyotime Biotechnology, China).

### 2.6. Lung wet-to-dry ratio

The mice were humanely euthanized, and their lungs were washed with cold PBS to remove blood stains. The initial lung weight, denoted as the wet weight (WW), was recorded, then dehydrated in a 60°C oven for a duration of 24 hours to ascertain the dry weight (DW). The ratio of lung wet weight to dry weight was then computed by dividing the WW by the DW, providing a metric of lung water content.

### 2.7. TUNEL assay

Tissue sections were treated with proteinase K for 25 minutes at 37°C, washed three times with PBS, and then stained with TUNEL solution (C1098, Beyotime, China) for 2 hours in a light-protected environment. After restaining with DAPI, the sections were examined under a fluorescence microscope (Leica, Germany).

### 2.8. Immunohistochemistry of HO-1 and NQO-1

Lung tissue sections were deparaffinized in xylene, followed by rehydration through a graded ethanol series, and placed in sodium citrate solution for 15 minutes at 98°C for antigen recovery. After blocking endogenous peroxidase with 3% H2O2, they were incubated with primary antibodies for HO-1 (10701-1-AP, Proteintech, China) and NQO-1 (A0047, Abconal, China) overnight at 4°C. After rewarming, they were incubated with the specific secondary antibody for 1 hour to amplify the signal, which was visualized using 3,3-diaminobenzidine tetrahydrochloride (DAB). Finally, the sections were stained with hematoxylin for 5 minutes to show the nuclei. Positive cells were quantified from five random fields per section under a light microscope.

### 2.9. Dihydroethidium (DHE) staining

For cellular ROS detection, treated Beas-2b cells were incubated with 5 μM DHE solution for 30 minutes at 37°C in the dark. For tissue ROS, fresh mouse lung tissues were embedded in OCT and sectioned into 8 μm frozen slices. The oxidized fluorescent dye DHE (HY-D0079, MCE, U.S.A.), was diluted to a ratio of 1:1000 and incubated with the sections for 30 minutes to assess ROS levels. Then, sections were counterstained with DAPI and examined under a fluorescence microscope. Fluorescence intensities were quantified using ImageJ software.

### 2.10. Cell viability assay

Cells were inoculated on a 96-well plate at a concentration of 5 × 103 cells/well and allowed to adhere and proliferate for 24 hours. Then, the cells were exposed to *t*-BHP and sAc15 for 24 hours. After adding a diluted CCK-8 reaction solution (HY-K0301, MCE, U.S.A.) to the cells, they were incubated at 37°C for 1.5 hours. The absorbance was quantified at 450 nm.

### 2.11. Western blotting

RIPA lysis buffer (89901, Thermo Fisher Scientific, U.S.A.), supplemented with 1% protease and phosphatase inhibitors (P1260, Epson, China), was employed for the extraction of proteins from cellular and tissue samples. Protein concentration was measured by the BCA Protein Assay Kit (23227, Thermo Fisher Scientific, U.S.A.). Fresh proteins were separated on SDS polyacrylamide gels, transferred to PVDF membranes via electrophoresis. After blocking with 5% skim milk, membranes were incubated overnight with primary antibodies, including anti-Nrf2 (A1244, Abconal, China), anti-HO-1 (10701-1-AP, Proteintech, China), anti-NQO-1 (Abconal, A0047, China), anti-Bcl-2 (sc-7382, Santa Cruz Biotechnology, CA), anti-Bax (ab32503, Abcam, UK), anti-Caspase3 (9662, CST, U.S.A.), anti-*β*-actin (AF7018, Affinity Biosciences, China), anti-Akt (2920S, CST, U.S.A.), and anti-phospho-Akt (4058S, CST, U.S.A.). The membranes were then incubated with secondary antibody (BL003A, Biosharp, China). Signal detection was carried out using an imaging system (iBright™ CL1500 Instrument, Thermo Fisher Scientific, U.S.A.) and quantified using ImageJ software.

### 2.12. Immunofluorescence staining

Lung tissue sections and cells were fixed with 4% solution of paraformaldehyde for 30 minutes to preserve their structure. Following permeabilization with 0.1% Triton X100 for 20 minutes, blocked with 1% BSA for 30 minutes, and incubated with specific primary antibodies Nrf2 (16396-1-AP, Proteintech, China), EpCAM (sc-66020, Santa Cruz, U.S.A.), and Cytochrome c (AC908, Beyotime Biotechnology, China) overnight at 4°C. Secondary antibodies with fluorescent markers were then applied for 1 hour. DAPI was applied for 10 minutes to stain the nuclei. Specifically, the localization of mitochondria was achieved through the utilization of Mito-Tracker Red CMXRos (C1035, Beyotime Biotechnology, China) before the staining and fixation of Cytochrome c. Subsequent image capture was carried out utilizing a Leica microscope (Germany).

### 2.13. Cellular ROS assay

Beas-2b cells were cultured in 96-well plates at a seeding density of 5*10^3^ cells per well and adhered for 24 hours. Following treatment with sAc15 and *t*-BHP according to the protocol provided with the Reactive Oxygen Species Detection Kit (S0033S, Beyotime, China), a medium containing 10 μM DCFH-DA and 10 μM Hoechst 33342 was added to the cells. This incubation was carried out at 37°C in a light-protected environment for 20 minutes. Image acquisition and analysis were conducted with the Operetta Harmony High-Content Imaging System version 4.8 (PerkinElmer).

### 2.14. JC-1 assay

The JC-1 assay kit (C2006, Beyotime Biotechnology, China) was utilized for the detection of alterations in MMP. Cells were cultivated in 96-well plates and treated with 10 mg/ml MitoProbe JC-1 at 37°C for a duration of 20 minutes. Variations in MMP were documented and imaged using an Operetta High Content Screening System (PerkinElmer).

### 2.15. Surface Plasmon resonance (SPR) assay

Real-time physical binding kinetics between sAc15 and recombinant Nrf2 protein were evaluated using a Biacore 1 K system (Cytiva) at 25°C. Recombinant human Nrf2 protein (IPD-X50094, Ipodix, 50 μg/mL in 10 mM sodium acetate buffer, pH 4.0) was immobilized onto a CM5 sensor chip (BR-1005-30, Cytiva) surface via standard amine coupling. The active channel was activated with a 1:1 mixture of 0.4 M EDC and 0.1 M NHS at 10 μL/min for 420 s, followed by ethanolamine blocking (1 M, pH 8.5). A protein-free reference channel was prepared under identical conditions for bulk background subtraction. Serial dilutions of sAc15 (0.156, 0.312, 0.625, 1.25, 2.5, 5, 10, 20 μM) in running buffer (1 × PBS-P + , pH 7.4, supplemented with 5% DMSO and 0.05% Tween-20) were injected over the chip surface. Association and dissociation phases were set to 100 s and 120 s, respectively, at a constant flow rate. The chip was regenerated using 10 mM glycine-HCl (pH 2.0) after each cycle. Kinetic rate constants (Ka, Kd) and equilibrium dissociation constants (KD) were derived by globally fitting double-referenced sensorgrams to a 1:1 Langmuir binding model and a steady-state affinity model using Biacore Evaluation Software.

### 2.16. Cell transfection with small interfering RNA (siRNA)

The NC-siRNA and Nrf2-siRNA were synthesized by GenePharma (Shanghai, China). Beas-2b cells were cultured in 6-well plates to 50% confluence. Transfection of si-Nrf2 or si-NC was performed using opti-MEM medium (Gibco, U.S.A.) and Lipofectamine 3000 (Invitrogen, U.S.A.). After 10 hours, the complete medium was replaced and the successfully transfected cells were further cultured for subsequent experiments. The relevant sequences are shown in the Supplementary Table1.

### 2.17. Mitochondrial superoxide assay

Cells were seeded in 96-well plates at 5000 cells/well and treated with sAc15 and t-BHP for the required time. A total of 5 μM MitoSOX Red probe (M36008, Invitrogen, U.S.A.) was added to the medium, and cells were incubated at 37°C for 30 minutes before two washes with prewarmed HBSS. Mitochondrial MitoSOX Red fluorescence was detected with a PerkinElmer high-content screening system, and data analysis was conducted using Operetta Harmony 4.8.

### 2.18. Flow cytometric analysis of cell apoptosis

After treatment, Beas-2b cells were digested with EDTA-free trypsin, washed twice with ice-cold PBS, and resuspended in 195 μL 1 × binding buffer. Then 5 μL Annexin V-FITC and 10 μL PI working solution (C1062L, Beyotime Biotechnology, China) were added. The suspension was gently vortexed and incubated in the dark at room temperature for 15 minutes. Samples were immediately detected on a flow cytometer (BD Biosciences, U.S.A.), and the proportion of apoptotic cells was calculated using BD Accuri software.

### 2.19. Statistical analysis

Experiments were conducted in triplicate or more, and the data were analyzed statistically using GraphPad Prism version 8.0. One-way ANOVA coupled with Tukey's post hoc test was applied for multiple comparisons, with a threshold of *p* < 0.05 to determine statistical significance.

### 2.20. Prediction of sAc15 and ALI-related targets

The GeneCards database (https://www.genecards.org/) was used to identify disease-related targets for ALI by searching the keyword ‘Acute lung injury.’ Targets with a relevance score above the median were selected for further analysis. The chemical structure of sAc15 was uploaded in SDF format to the SwissTargetPrediction database (https://www.swisstargetprediction.ch/) and PharmMapper database (https://www.lilab-ecust.cn/pharmmapper/) to predict potential drug targets. Gene names were standardized by retrieving gene data from the UniProt database (https://www.uniprot.org/).

### 2.21. Screening of intersection targets

The predicted drug targets of sAc15 and disease-associated targets of ALI were consolidated, and their intersection targets were identified. A Venn diagram illustrating the overlapping targets was generated using the online tool Jvenn (https://jvenn.toulouse.inra.fr/).

### 2.22. Construction of protein-protein interaction (PPI) network

Intersection targets were imported into the STRING database (https://cn.string-db.org/) with a minimum required interaction score set at 0.4. Single nodes without interactions were omitted. The resulting PPI network was visualized using Cytoscape software version 3.10.2. The CytoNCA plugin was employed to calculate degree centrality (DC) values for each node, enabling the identification and ranking of core targets based on their DC scores.

### 2.23. Gene Ontology (GO) functional annotation and Kyoto Encyclopedia of Genes and Genomes (KEGG) pathway enrichment analysis

The intersecting targets were uploaded to the DAVID database (https://david.ncifcrf.gov/) to perform GO functional annotation and KEGG pathway enrichment analysis. This approach facilitated the association of targets with biological functions and signaling pathways. Enrichment results with statistical significance (*P* < 0.05) were selected and visualized for interpretation.

### 2.24. Molecular docking

The three-dimensional structure of sAc15 was obtained from the PubChem database (https://pubchem.ncbi.nlm.nih.gov/), while PI3K (PDB ID: 8EXL), AKT1 (PDB ID: 7NH5), Nrf2 (PDB ID: 8XGK) crystal structure was acquired from the UniProt database (https://www.uniprot.org/). Both structures underwent optimization through the removal of water molecules and the addition of hydrogen atoms. The ligand-binding active sites were identified using AutoDockTools 1.5.7, followed by molecular docking simulations with AutoDock Vina. The resulting complexes were subsequently refined in PyMOL, which enabled visualization of key interaction patterns including amino acid residues, hydrogen bonds, and other intermolecular contact sites.

## 3. Results

### 3.1. sAc15 attenuates LPS-induced ALI in mice

In the model of LPS-induced ALI in mice, lung tissues from each experimental group underwent H&E staining for histopathological evaluation. As shown in Figure 1C, the alveolar structure in both the Vehicle and sAc15 groups remained intact, characterized by distinct interstitial spaces, with only a minimal presence of inflammatory cells observed in the alveolar septa of the sAc15 group. In contrast, the LPS group exhibited significantly thickened alveolar septa, extensive infiltration of inflammatory cells, and the presence of light pink exudates within the alveolar spaces, indicative of pulmonary edema. Importantly, mice pretreated with sAc15 (sAc15+LPS group) showed significantly alleviated lung histopathological damage and preserved alveolar structure, with lung injury scores substantially lower than those in the LPS group (Figure 1D). Subsequent assessment of pulmonary edema revealed that sAc15 effectively decreased the lung wet-to-dry weight ratio and total protein concentration in BALF induced by LPS, suggesting an improvement in lung permeability and edema (Figure 1E–F). Additionally, analysis of inflammatory cytokines revealed that LPS administration significantly elevated the levels of IL-1β, TNF-*α*, and IL-6 in BALF, whereas their expression was markedly attenuated in the sAc15+LPS group (Figure 1G–I). Taken together, these results suggest that sAc15 pretreatment mitigates pathological damage and inflammatory responses in LPS-induced ALI in mice.

### 3.2. sAc15 prevents oxidative stress and apoptosis in lung tissues of LPS-induced ALI mice

**Figure 2.**
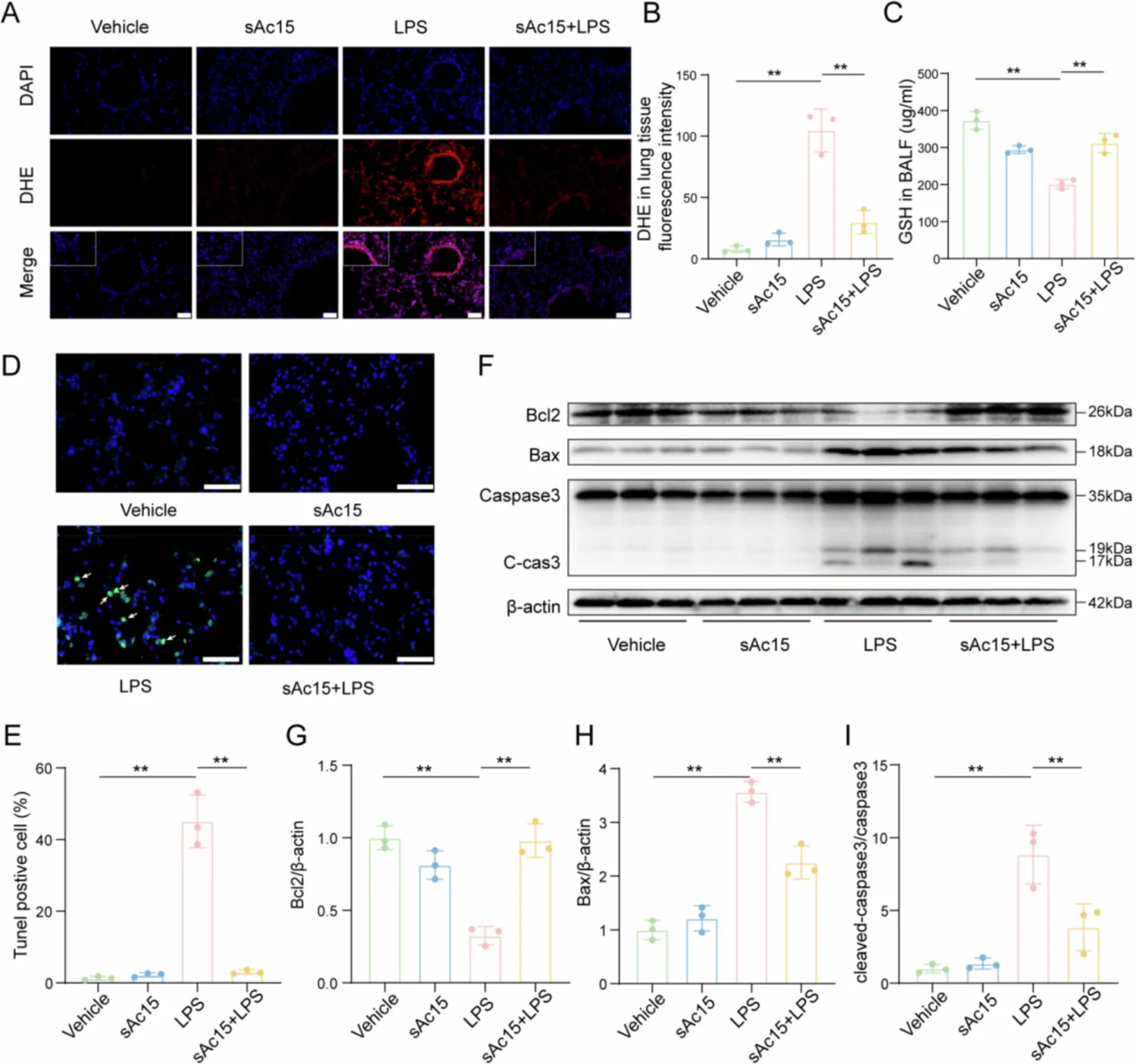
sAc15 prevents LPS-induced oxidative stress and cell apoptosis in mice lung tissue. Mice were pretreated with sAc15 (20 mg/kg, 1 hour) before intratracheal LPS instillation (10 mg/kg) for 24 hours. (A–B) The generation of ROS in lung tissue was evaluated by employing fluorescent-labeled DHE staining. Scale: 20 μm. (C) GSH activity in BALF was measured. (D) Lung tissues were fluorescent stained with TUNEL and positive cells are indicated by arrowheads. Scale: 20 μm. (E) Number of positive cells in lung tissue. (F–I) The levels of apoptosis-related proteins were assessed using western blotting. Data are presented as means ± SEM (*n* = 3). *P < 0.05, **P < 0.01.

ROS levels in frozen lung tissue sections were assessed by DHE staining. The findings indicated that treatment with sAc15 alone did not significantly alter ROS levels compared to the Vehicle group. Conversely, exposure to LPS markedly elevated ROS generation in alveolar epithelial cells and adjacent cells, an effect that was substantially inhibited by pretreatment with sAc15 (Figure 2A–B). In alignment with the DHE staining results, sAc15 pretreatment effectively prevented the depletion of the antioxidant enzyme GSH, which is essential for protection against LPS-induced oxidative stress (Figure 2C). Given that apoptosis is a major outcome of oxidative damage, we further investigated the effect of sAc15 on pulmonary cell apoptosis. Quantitative analysis of apoptotic cells by TUNEL staining revealed no significant difference in TUNEL-positive cell numbers between the Vehicle and sAc15 groups under normal conditions. However, LPS administration significantly increased apoptotic cell counts in lung tissues, whereas sAc15 pretreatment markedly reduced LPS-induced apoptosis (Figure 2D–E). Western blotting analysis provided additional confirmation that LPS stimulation upregulated the expression of pro-apoptotic proteins Bax and cleaved-caspase3, while downregulating the anti-apoptotic protein Bcl-2. These molecular alterations were significantly reversed following pretreatment with sAc15 (Figure 2F–I). These findings demonstrate that sAc15 pretreatment effectively alleviates LPS-induced oxidative stress and apoptosis in mouse lung tissues.

### 3.3. sAc15 alleviates *t*-BHP-induced oxidative damage and mitochondrial dysfunction in Beas-2b cells

**Figure 3.**
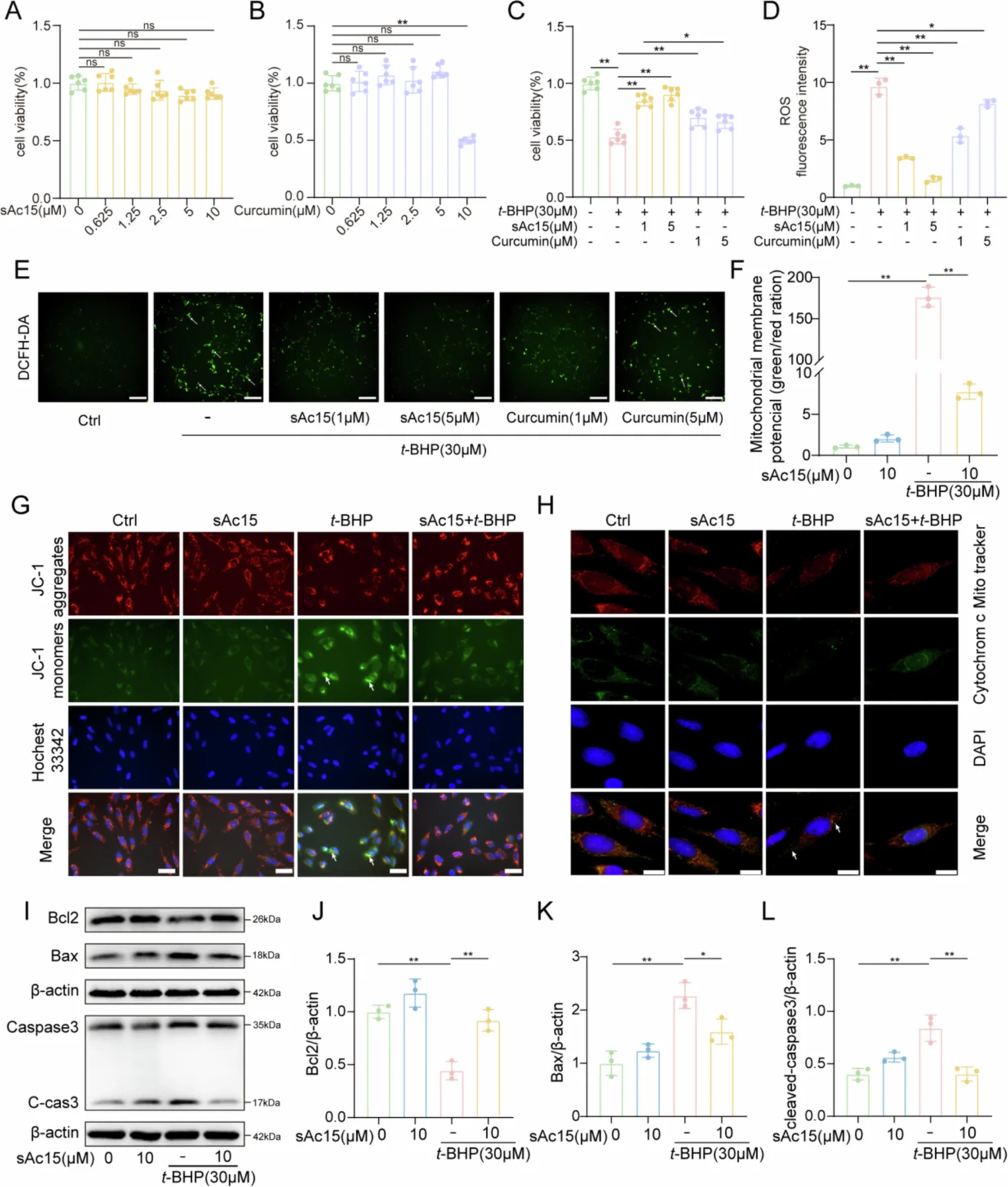
sAc15 alleviates t-BHP-induced oxidative damage and mitochondrial dysfunction in Beas-2b cells. (A) Intrinsic cytotoxicity of gradient sAc15 (0–10 μM, 24 hours) assessed by CCK-8 assay. (B) Intrinsic cytotoxicity of gradient curcumin (0–10 μM, 24 hours) assessed by CCK-8 assay. Beas-2b cells were pretreated with indicated concentrations of sAc15 or curcumin for 1 hour prior to exposure to 30 μM *t*-BHP for 24 hours. (C) Effect of sAc15 or curcumin on *t*-BHP induced cytotoxicity was determined by CCK8 kit. (D–E) Impact of sAc15 or curcumin on *t*-BHP inspired ROS formation in Beas-2b. Scale: 200 μm. (F–G) Influence of sAc15 on *t*-BHP depleted MMP as determined by JC-1 staining. Scale: 50 μm. (H) Co-localized fluorescent staining of Cytochrome c (green) and Mito Tracker (red). Scale: 20 μm. (I–L) Proteins associated with apoptosis were evaluated by Western blotting. All results were expressed as means ± SEM of three independent experiments. *P < 0.05, **P < 0.01.

sAc15 is a synthetic derivative obtained by modifying the unstable *β*-diketone moiety of natural curcumin. We first evaluated the intrinsic cytotoxicity of both compounds and found that neither exhibited significant toxicity to Beas-2b epithelial cells at concentrations up to 5 μM. However, when the concentration reached 10 μM, natural curcumin showed a marked increase in cytotoxicity, whereas sAc15 maintained a highly favorable safety profile (Figure 3A–B). To assess functional protection, we utilized 30 μM *t*-BHP, a well-established oxidative stress inducer, to simulate oxidative injury in lung epithelial cells. CCK-8 assays demonstrated that pretreatment with either curcumin or sAc15 attenuated *t*-BHP induced cytotoxicity in a dose-dependent manner. Notably, sAc15 conferred significantly greater cytoprotective efficacy than curcumin at equivalent doses (Figure 3C). Consistently, ROS fluorescence staining revealed that sAc15 robustly suppressed *t*-BHP induced intracellular ROS accumulation in a concentration-dependent manner. Although parent curcumin also displayed inherent antioxidant capacity, its activity was substantially weaker than that of sAc15. Furthermore, at elevated concentrations, the cytotoxicity of curcumin progressively outweighed its antioxidant benefits (Figure 3D–E). Furthermore, delayed sAc15 administration at 1 hour or 2 hours post-*t*-BHP challenge significantly rescued cell survival and attenuated ROS accumulation, establishing an effective 2 hours post-injury therapeutic window (Fig. S1 A–C). Collectively, these findings demonstrate that structural optimization of sAc15 not only minimizes inherent cytotoxicity but also enhances cytoprotective antioxidant efficacy. Even when administered after injury, it still exhibits effective therapeutic protective effects.

Given that mitochondria are the primary sites of cellular ROS production, mitochondrial function in Beas-2b cells was assessed by monitoring MMP changes. As shown in Figure 3F–G, the ratio of green to red fluorescence, indicative of MMP, was significantly increased in the *t-BHP-*treated group compared to the control and sAc15 groups, suggesting mitochondrial dysfunction. Notably, pretreatment with sAc15 effectively restored MMP levels. Moreover, immunofluorescence co-localization staining revealed the interaction between mitochondria and Cytochrome c. Exposure to *t*-BHP prompted the release of Cytochrome c from mitochondria into the cytoplasm, whereas sAc15 markedly inhibited this translocation (Figure 3H). Western blotting analysis further corroborated alterations in apoptosis-related proteins. Consistent with the in vivo findings, sAc15 pretreatment reversed the *t*-BHP-induced upregulation of pro-apoptotic Bax and cleaved-caspase3, as well as the downregulation of anti-apoptotic Bcl-2 (Figure 3I–L). These results indicate that sAc15 effectively ameliorates *t*-BHP-induced oxidative damage and mitochondrial dysfunction in Beas-2b cells.

### 3.4. Network pharmacology analysis of sAc15 in the treatment of ALI and its regulatory effects on the PI3K/AKT signaling pathway

**Figure 4.**
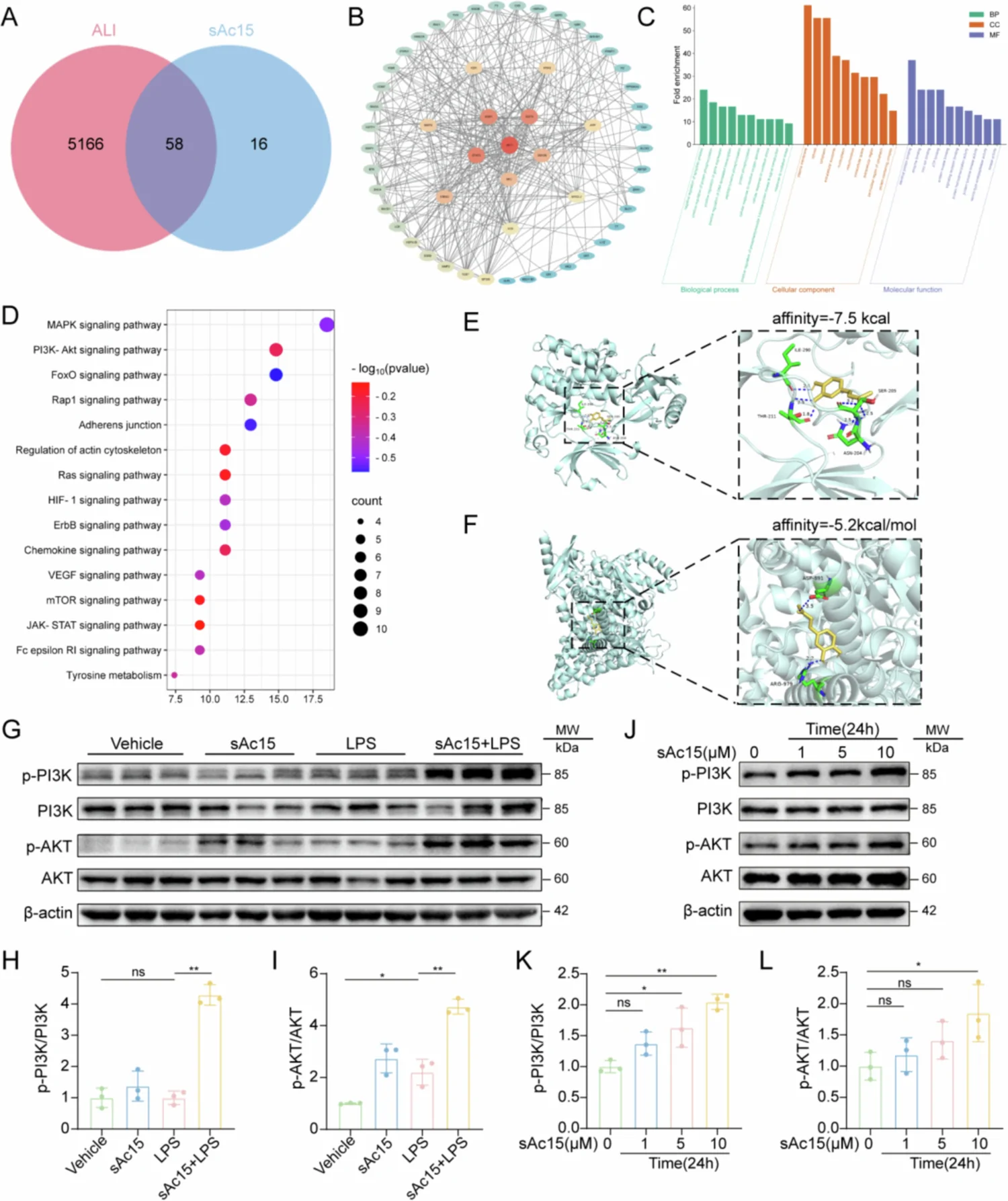
Network pharmacology analysis of sAc15 in the treatment of ALI and its regulatory effects on the PI3K/AKT signaling pathway. (A) Potential targets of sAc15 for treating ALI. (B) Construction of protein-protein interaction (PPI) network and identification of key targets. (C) Top 10 enriched terms in GO analysis including BP, CC, and MF. (D) Top 15 pathways identified by KEGG enrichment analysis. (E–F) Molecular docking models of sAc15 with AKT1 and PI3K, respectively. (G–I) The levels of PI3K/AKT signaling pathway were detected by western blotting in lung tissue of mice. (J–L) Western blotting analysis showing the effects of different concentrations of sAc15 on the PI3K/AKT signaling pathway. All results were expressed as means ± SEM of three independent experiments. *P < 0.05, **P < 0.01.

To elucidate the potential molecular mechanisms underlying the therapeutic effects of sAc15 on ALI, network pharmacology approaches were employed. Initially, a total of 5,244 ALI-related disease targets were intersected with 74 potential molecular targets of sAc15, resulting in the identification of 58 overlapping targets, as depicted in a Venn diagram (Figure 4A). Subsequently, a topological analysis based on DC was conducted to rank these overlapping targets, facilitating the construction of PPI network and the identification of core proteins, including AKT1, STAT3, EGFR, ESR1, GSK3B, and SRC (Figure 4B). GO enrichment analysis of the overlapping targets demonstrated significant biological functions across three categories. Biological process (BP) analysis highlighted terms such as negative regulation of apoptotic process, positive regulation of protein phosphorylation, cellular response to oxidative stress, and positive regulation of phosphatidylinositol 3-kinase/protein kinase B (PI3K/AKT) signal transduction (Figure 4C). KEGG pathway enrichment analysis indicated that the molecular mechanism by which sAc15 exerts therapeutic effects on ALI may involve the PI3K-AKT signaling pathway, Rap1 signaling pathway, and Ras signaling pathway, among others (Figure 4D). Following the results from GO and KEGG enrichment analyzes, molecular docking was performed between sAc15 and the core targets AKT1 and PI3K. The docking simulations revealed that sAc15 binds to AKT1 at residues ILE-290, THR-211, ASN-204, and SER-205 with a binding energy of −7.56 kcal/mol (Figure 4E). In the case of PI3K, sAc15 binds at ASP-891 and ARG-979 with a binding energy of −5.2 kcal/mol (Figure 4F). To further substantiate these findings, we investigated alterations in PI3K/AKT levels within mouse lung tissue. The results indicated that pretreatment with sAc15 resulted in a modest upregulation of *p*-PI3K and *p*-AKT levels, thereby enhancing the activation of the PI3K/AKT pathway during the acute inflammatory phase and subsequently inhibiting cell apoptosis (Figure 4G–I). Concurrently, Beas-2b cells were exposed to varying concentrations of sAc15, revealing a dose-dependent increase in *p*-PI3K and *p*-AKT levels (Figure 4J–L). These observations suggest that the activity of sAc15 is preliminarily linked to the modulation of the PI3K/AKT signaling pathway.

### 3.5. sAc15 activates the Nrf2 signaling pathway and its downstream antioxidant proteins

**Figure 5.**
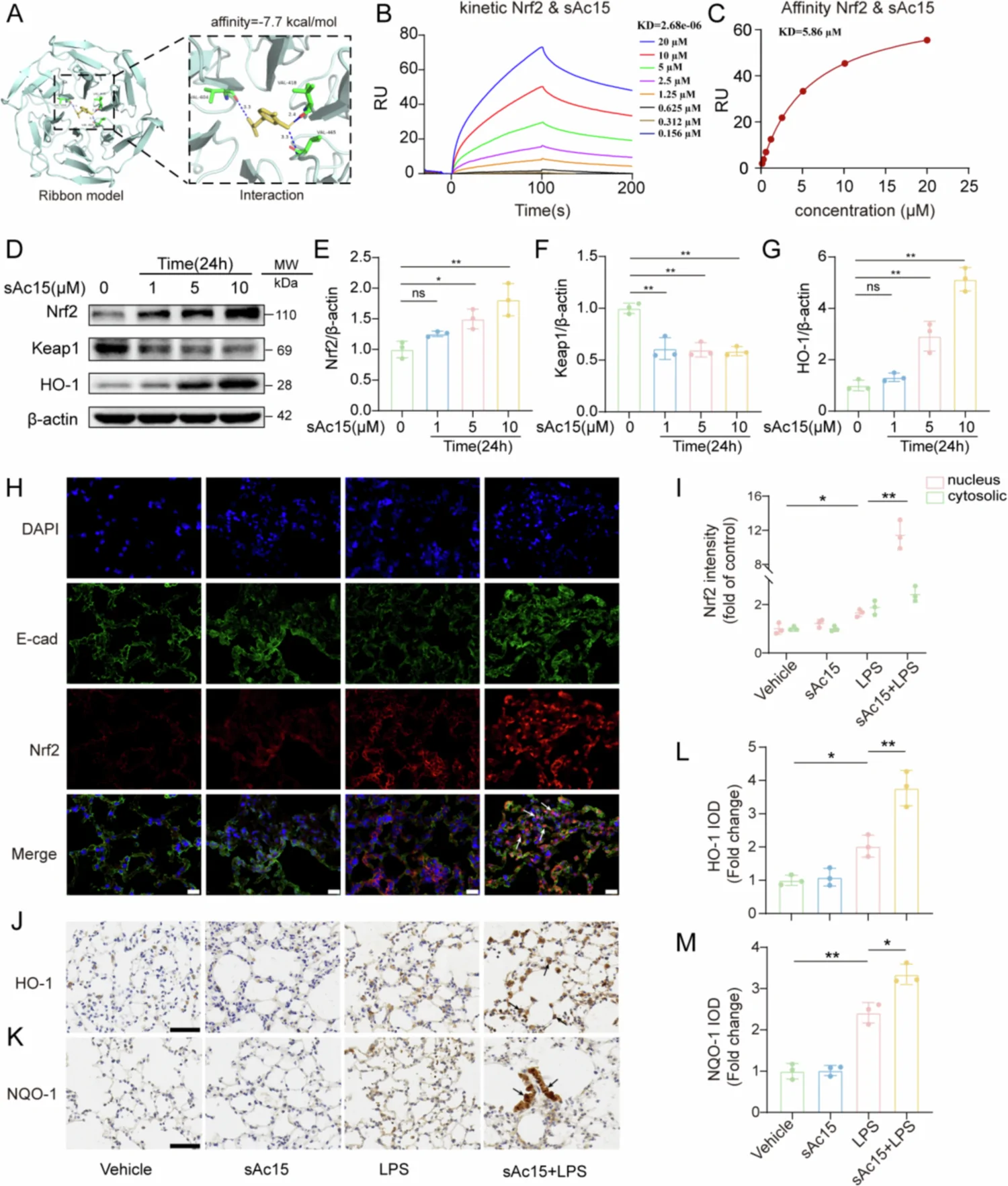
sAc15 activates Nrf2 signaling pathway and its downstream antioxidant proteins. (A) Molecular docking analysis of the sAc15-Nrf2 binding interface and Key hydrogen bond interactions between sAc15 and Nrf2 residues VAL-418, VAL465, and VAL-604. (B) Surface Plasmon resonance (SPR) analysis of sAc15 and recombinant Nrf2. sAc15 was added at different concentrations ranging from 0.156 μM to 20 μM. KD = 2.68 μM. (C) The steady-state binding curve of sAc15 and Nrf2. KD = 5.86 μM. (D–G) Western blot analysis and quantitative analysis of Nrf2, Keap1, and HO-1 protein levels in Beas-2b cells treated with gradient concentrations of sAc15 (1, 5, 10 μM) for 24 hours. (H–I) Immunofluorescence staining of mice lung tissue reveals the appearance and location of Nrf2 in lung epithelial cells. Scale: 20 μm (J–M) Immunohistochemical results of the expression of HO-1 and NQO-1. Scale: 50μm. Data are expressed as mean ± SEM from three independent experiments. *P < 0.05, **P < 0.01.

Nrf2 is a key transcription factor involved in cellular antioxidant defense. We investigated the regulatory effect of sAc15 on Nrf2. Molecular docking simulations demonstrated that sAc15 has a strong binding affinity for Nrf2, with a binding free energy of –7.7 kcal/mol (Figure 5A). Surface Plasmon resonance (SPR) analysis further confirmed the direct interaction between the recombinant sAc15 and Nrf2, with the dissociation constant (KD) being 2.68 μM. The steady-state affinity analysis revealed a clear saturation trend with steady-state KD of 5.86 μM. With he low micromolar range, both kinetic and steady-state KD values provides unambiguous physical proof that Nrf2 is a primary direct binding target of sAc15 (Figure 5B–C). In vitro experiments demonstrated that treatment with sAc15 at varying doses upregulated the protein expression of Nrf2 and its downstream antioxidant enzymes, accompanied by the accelerated degradation of Keap1, a negative regulator of Nrf2 (Figure 5D–G). Notably, at equivalent concentrations, natural curcumin induced only a modest upregulation of Nrf2 and HO-1 expression (Fig. S2A-D). These comparative findings further reinforce that sAc15 possesses a substantially higher efficacy than parent curcumin in modulating the cellular antioxidant defense system. Immunofluorescence staining of mouse lung tissues showed that sAc15 moderately increased Nrf2 expression, although this change was not statistically significant compared with the Vehicle group. LPS stimulation induced a slight upregulation of Nrf2, which was further enhanced by sAc15 pretreatment. Notably, sAc15 pretreatment also facilitated the nuclear translocation of Nrf2, thereby activating downstream antioxidant transcription factors (Figure 5H–I). Subsequently, immunohistochemical analysis was performed to assess the expression of key Nrf2 downstream antioxidant enzymes, HO-1 and NQO-1. The results revealed that sAc15 pretreatment enhanced the modest upregulation of HO-1 and NQO-1 induced by LPS (Figure 5J–M). Taken together, these findings indicate that the antioxidant mechanism of sAc15 is linked to the activation of the Nrf2 signaling pathway and its downstream antioxidant proteins.

### 3.6. sAc15 reduces oxidative damage by activating the PI3K/AKT/Nrf2 pathway

**Figure 6.**
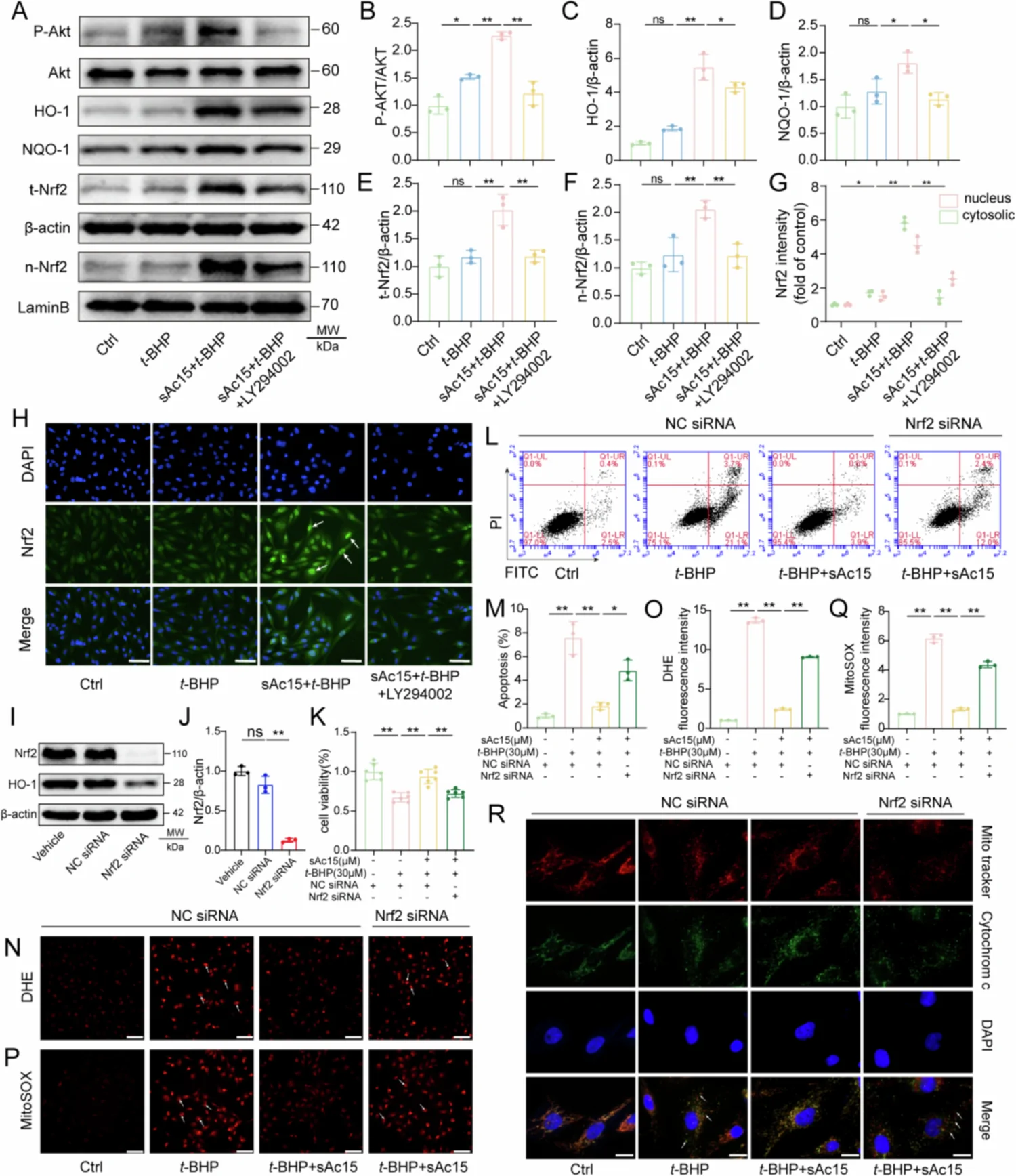
sAc15 mediates PI3K/AKT/Nrf2 signaling pathway in Beas-2b cells. Beas-2b cells were pretreated with the PI3K inhibitor LY294002 (5  μM, 1 hour) or transfected with Nrf2 siRNA before sAc15 (10 μM, 1 hour) and *t*-BHP (30 μM, 24 hours) challenge. (A−F) Western blot analysis of *p*-AKT, Nrf2, HO-1, and NQO-1 expression levels in cells pretreated with LY294002. (G−H) LY294002 inhibits sAc15-regulated Nrf2 translocation to the nucleus as observed by immunofluorescence staining. Scale: 50 μm. (I−J) Western blot validation of si-Nrf2 knockdown efficiency in Beas-2b cells. (K) After knocking down Nrf2, the protective effect of sAc15 against the reduction in cell viability caused by *t*-BHP was detected by the CCK8 assay kit. (L−M) Flow cytometry was used to detect the proportion of apoptotic cells within each group. (N−O) The accumulation of intracellular reactive oxygen species (ROS) was detected by DHE staining. Scale: 100 μm. (P−Q) Quantitative analysis of mitochondrial superoxide levels evaluated by MitoSOX Red staining. Scale: 100 μm. (R) After knocking down Nrf2, the inhibitory effect of sAc15 on the release of cytochrome C from mitochondria was detected by fluorescence co-localization. Scale: 20 μm. Data are presented as mean ± SEM from three independent experiments. *P < 0.05, **P < 0.01.

Changes in the PI3K/AKT/Nrf2 signaling pathway were evaluated by Western blotting and immunofluorescence. Results showed that exposure to *t*-BHP slightly increased the expression levels of *p*-AKT, total Nrf2, nuclear Nrf2, HO-1, and NQO-1, whereas treatment with sAc15 further enhanced these expression levels (Figure 6A–F). Immunofluorescence analysis further corroborated that sAc15 not only upregulated overall Nrf2 expression but also promoted its nuclear translocation, consistent with the in vivo findings. The application of the PI3K/AKT-specific inhibitor LY294002 partially attenuated the sAc15-induced enhancement of Nrf2 expression, nuclear translocation, and the upregulation of downstream antioxidant enzymes (Figure 6G–H). These results indicated that sAc15-mediated activation of Nrf2 depends on the PI3K/AKT signaling pathway. To further elucidate the pivotal role of Nrf2 in the antioxidant mechanism of sAc15, targeted gene silencing was performed using Nrf2-specific siRNA in Beas-2b cells, with knockdown efficiency validated by Western blotting (Figure 6I–J). Functionally, Nrf2 depletion significantly diminished the protective effect of sAc15 against *t*-BHP-induced oxidative damage, as evidenced by decreased cell viability (Figure 6K), restored cell apoptosis (Figure 6L–M), elevated intracellular ROS accumulation (Figure 6N–O), and heightened mitochondrial superoxide levels (Figure 6P–Q). Additionally, immunofluorescence analysis revealed that Nrf2 knockdown attenuated the ability of sAc15 to suppress the *t*-BHP-induced release of Cytochrome c from mitochondria to the cytoplasm (Figure 6R). These findings provide compelling evidence that sAc15 exerts its antioxidant protective effects through the PI3K/AKT/Nrf2 signaling axis.

## 4. Discussion

ALI and its severe form, ARDS, remain life-threatening clinical entities characterized by uncontrolled oxidative stress, alveolar-capillary barrier disruption, and widespread epithelial cell damage [22]. While natural curcumin possesses potent antioxidant capacity, its clinical application is fundamentally bottlenecked by poor chemical stability and rapid pro-oxidant shift at micromolar doses [23,24]. In the present study, we demonstrated that sAc15, a novel structurally optimized monoenone-monocarbonyl curcumin analog, robustly protects against LPS and *t*-BHP induced pulmonary injury both in vivo and in vitro. Crucially, through biophysical characterization and genetic loss-of-function approaches, we established that sAc15 directly engages the master redox regulator Nrf2 (KD = 2.68 μM) and activates the upstream PI3K/AKT signaling cascade, thereby restoring mitochondrial integrity, mitigating apoptosis, and quenching excessive intracellular ROS.

Numerous studies have reported that natural products and traditional Chinese medicine extracts, such as flavonoids, alkaloids, and terpenoids, exhibit potent anti-inflammatory and antioxidant activities [25]. Nevertheless, their clinical translation remains severely hampered by common drawbacks, including poor chemical stability, low bioavailability, and ill-defined mechanisms. Natural curcumin is no exception, as its highly unstable *β*-diketone moiety renders it prone to rapid metabolic degradation [16]. To overcome these limitations, sAc15 was purposefully designed by removing one double bond and one aromatic ring from the dienone backbone, achieving superior chemical stability and attenuated intrinsic cytotoxicity while retaining the essential *α*, *β*-unsaturated ketone scaffold [18]. In line with this structural optimization, our in vitro evaluation demonstrated that sAc15 displayed significantly lower cytotoxicity than parent curcumin at concentrations up to 10 μM, while simultaneously exerting superior antioxidant efficacy at equivalent molar doses.

The robust antioxidant efficacy of sAc15 stems from a ‘dual-action’ antioxidant framework conferred by its retained phenolic hydroxyl groups and the *α*, *β*-unsaturated ketone moiety. Phenolic moieties in curcuminoids directly neutralize ROS via hydrogen atom transfer (HAT) or single-electron transfer (SET) mechanisms [26,27]. In this study, sAc15 manifested promising protective effects against LPS-induced lung injury. Our findings indicate that sAc15 significantly mitigated LPS-induced pulmonary tissue injury and edema, concurrently attenuating ROS accumulation and preserved BALF GSH levels in ALI mice, thereby augmenting in vivo ROS scavenging. In vitro, we employed *t*-BHP to simulate the ROS-rich milieu to which epithelial cells are subjected, and our data revealed that sAc15 effectively inhibited ROS production in epithelial cells challenged by *t*-BHP.

Concurrently, the electrophilic *α*, *β*-unsaturated carbonyl moiety can alkylate reactive cysteine residues on Keap1, triggering the dissociation and nuclear translocation of Nrf2 to drive Nrf2/ARE-dependent transcriptional activation of endogenous antioxidant defenses [28]. SPR assays provided biophysical evidence that sAc15 directly binds to Nrf2 protein in a concentration-dependent manner, with kinetic and steady-state affinity analyzes jointly supporting a low-micromolar binding interaction (KD = 2.68μM). By orchestrating direct radical quenching with sustained, genome-level activation of antioxidant targets [10], sAc15 confers a clear therapeutic advantage over conventional antioxidants that rely solely on short-lived, stoichiometric ROS scavenging. As this study shows, sAc15 modestly elevated Nrf2 levels in mice lung tissues and potentiated the protective upregulation of Nrf2 in response to LPS. Moreover, sAc15 facilitated Nrf2 nuclear translocation, initiated the transcription and translation of its target downstream antioxidant proteins HO-1 and NQO-1, thereby enhanced antioxidant enzymes expression in vivo.

Beyond direct interaction with Nrf2, sAc15 further fortifies its antioxidant capacity by activating upstream modulators of Nrf2. A variety of natural compounds derived from traditional medicines, such as rosavidin and sipeimine, have been reported to confer organ-protective effects via PI3K/AKT related pathways [29,30]. This suggests that the PI3K/AKT axis represents a convergent stress adaptive hub engaged by structurally diverse phytochemicals. Particularly in pulmonary pathologies, activation of the PI3K/AKT pathway protects lung tissues against hyperoxia-induced injury by upregulating Nrf2 expression [31], and its activation has similarly been shown to attenuate smoke inhalation-induced lung injury [32]. Mechanistically, PI3K/AKT signaling is intimately linked to Nrf2 activation [33], wherein the downstream phosphorylation cascade facilitates the nuclear translocation and transcriptional activation of Nrf2 [31,32]. Intriguingly, the PI3K/AKT pathway was prioritized as a primary target in our network pharmacology analysis. Molecular docking simulations provided hypothesis-generating support, revealing favorable binding energies of sAc15 with AKT1 (−7.56 kcal/mol) and PI3K (−5.2 kcal/mol). Consistently, sAc15 treatment significantly upregulated PI3K/AKT phosphorylation levels both in murine lung tissues and in pulmonary epithelial cells. Crucially, pharmacological inhibition of PI3K/AKT blunted sAc15-mediated Nrf2 activation and nuclear accumulation, subsequently dampening the induction of downstream antioxidant enzymes.

Alveolar epithelial cells constitute the primary structural and functional barrier of the alveolar-capillary membrane, and their severe dysfunction and apoptosis represent central drivers in the pathological progression of acute lung injury (ALI) [3]. Consequently, the present study focused on the apoptotic fate of lung epithelial cells. Excessive accumulation of ROS impairs mitochondrial membrane permeability and dissipates the MMP, triggering the mitochondrial release of Cytochrome c, mitochondrial DNA, and pro-apoptotic factors that ultimately activate the intrinsic apoptotic cascade [34,35]. As mitochondria also serve as the principal intracellular factory for ROS production, growing evidence indicates that PI3K/AKT phosphorylation activates Nrf2 to attenuate superoxide generation and confer mitochondrial preservation [36–38]. Consistently, our findings demonstrate that sAc15 plays a key role in rescuing mitochondrial dysfunction; by activating the PI3K/AKT signaling axis, sAc15 effectively reverses *t-*BHP-induced MMP loss, restricts the cytosolic leakage of cytochrome c, and suppresses epithelial cell apoptosis. Given that lung injury encompasses complex pathological cascades including macrophage-mediated neutrophil recruitment driving inflammatory storms as well as non-apoptotic cell death modes in epithelial cells, future investigations will systematically unravel the cell-type-specific actions of sAc15, thereby laying a solid preclinical foundation for ultimate clinical translation.

## 5. Conclusion

**Figure 7.**
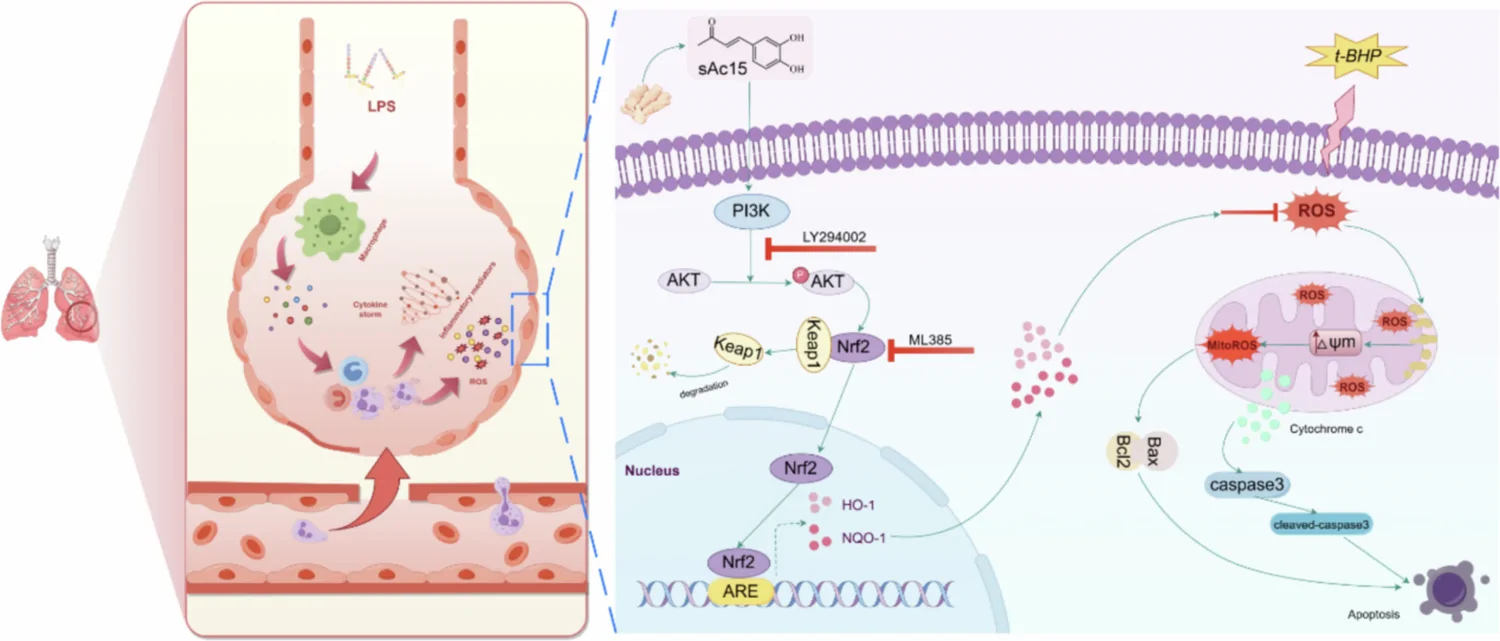
The diagram of underlying mechanisms of the sAc15 protects against LPS-induced acute lung injury. This picture was generated by Figdraw.

In this study, we demonstrated that the novel curcumin analog sAc15 attenuates mitochondrial damage and apoptosis caused by accumulation of ROS, ameliorating LPS-induced injury in lung epithelial cells. This protective effect may be mediated by activation of the PI3K/AKT/Nrf2 signaling axis (Figure 7). Our results propose sAc15 as a promising therapeutic agent for the management of ALI.

## Supplementary Material

ARRIVE Author Checklist.pdf ARRIVE Author Checklist.pdf

Supplementary materials.docx Supplementary materials.docx

## Data Availability

All supporting the findings of this report are included in this article. The data and materials are fully available upon reasonable request.
