# Supplementary material for: A novel curcumin analog sAc15 alleviates LPS-induced acute lung injury by activating the PI3K/AKT/Nrf2 pathway in pulmonary epithelial cells
Source: Redox Rep. 2026 Aug 22;31(1):2721883. doi: 10.1080/13510002.2026.2721883 (PMC13501739; doi:10.1080/13510002.2026.2721883)
Supplement: Supplementary file 2 [file YRER_A_2721883_SM6899.docx]

**Supplementary materials**

Supplementary Table 1.

| Gene | Oligo Sequence |
| --- | --- |
| Negtive control | 5’-UUCUCCGAACGUGUCACGUTT-3’ |
| NFE2L2 | 5’-GCACCUUAUAUCUCGAAGUTT-3’ |

**Fig.S1 Post-injury therapeutic efficacy of sAc15 in *t*-BHP challenged Beas-2b cells. (A)** Cell viability determined by CCK-8 assay in Beas-2b cells post-treated with sAc15 for 24 h following *t*-BHP exposure at indicated time intervals (1, 2, 3, or 4 h). **(B-C)** Representative fluorescence images and quantitative analysis of intracellular ROS levels detected by DCFH-DA staining in Beas-2b cells treated with sAc15 for 24h after 1h or 2h of *t*-BHP insult. Scale: 100μm. The means and SEM of three studies were used to express all results. *P <0.05, **P< 0.01.

**Fig.S2 Comparison of Nrf2 signaling pathway activation between curcumin and sAc15 in Beas-2b cells.** **(A)**The expression levels of Nrf2, HO-1 and Keap1 in Beas-2b cells treated with gradient concentrations (1, 5 μM) of curcumin or sAc15 for 24h. **(B–D)** Quantitative statistical analysis of relative protein levels of Nrf2, HO-1 and Keap1. The means and SEM of three studies were used to express all results. *P <0.05, **P< 0.01.
